# Clinical and Genetic Characteristics of 18 Patients from Southeast China with ABCA4-Associated Stargardt Disease

**DOI:** 10.3390/ijms26073354

**Published:** 2025-04-03

**Authors:** Xinyu Liu, Zehao Liu, Jinli Cui, Chen Tan, Wenmin Sun, Ying Lin

**Affiliations:** 1 State Key Laboratory of Ophthalmology, Guangdong Provincial Key Laboratory of Ophthalmology and Visual Science, Zhongshan Ophthalmic Center, Sun Yat-sen University, Guangzhou 510060, China; liuxy677@mail2.sysu.edu.cn (X.L.); liuzh228@mail2.sysu.edu.cn (Z.L.); bycuijinli@163.com (J.C.); sunwenmin@mail.sysu.edu.cn (W.S.); 2 Zhongshan School of Medicine, Sun Yat-sen University, Guangzhou 510080, China; tanch28@mail2.sysu.edu.cn

**Keywords:** Stargardt disease, ABCA4 gene, next-generation sequencing, mutation, allelic heterogeneity

## Abstract

Stargardt disease (STGD1), the most common retinal dystrophy caused by pathogenic variants of the biallelic *ABCA4* gene, results in irreversible vision loss. This cross-sectional case series study analyzes 18 unrelated Stargardt disease (STGD1) patients from southeast China, examining clinical and genetic features. Ophthalmological assessments included BCVA, ophthalmoscopy, fundus photography, and autofluorescence, with ultra-widefield OCT angiography carried out on one patient. Genetic testing uses targeted exome sequencing for eye disease genes. The mean age of onset was 44.3 years for adult onset (6 patients) and 9.6 years for childhood/adolescent onset (12 patients). The mean logMAR visual acuity was 0.96 (right eye) and 0.91 (left eye). Eight novel *ABCA4* variants were found, including two nonsense, two frameshift deletions, one copy number variant, one splice-site alternation, and two deep intronic variants. The genotypes are as follows: 77.8% (14/18) biallelic heterozygous, 16.7% (3/18) homozygous, and one patient with three variants. The study underscores STGD1’s phenotypic and genotypic diversity, expands the ABCA4 mutation spectrum, and offers insights into therapeutic strategies.

## 1. Introduction

Stargardt disease (STGD1; OMIM 248200) is the most prevalent form of inherited retinal dystrophy, with a prevalence of 1:8000 to 1:10,000 [1,2]. First described by Karl Stargardt in 1909, this disease typically manifests as central vision loss in childhood or adolescence, although late-onset forms in adulthood are also observed. The hallmark clinical features of STGD1 include the presence of macular atrophy and yellow-white flecks at the level of the retinal pigment epithelium (RPE) at the posterior pole [3].

Mutations in the *ABCA4* gene, located on chromosome 1 [4], are the primary cause of Stargardt disease, which follows an autosomal recessive inheritance pattern. In addition, autosomal dominant mutations in *ELOVL4* (STGD3; OMIM 600110) on chromosome 6 and *PROM1* mutations on chromosome 4 (STGD4; OMIM 603786) can also lead to the development of Stargardt-like disease. To date, over 4000 *ABCA4* variants have been identified, contributing to the significant clinical and genetic heterogeneity of the disease. The biallelic *ABCA4* variants cause a group of *ABCA4*-related retinopathies, including Stargardt disease (STGD1) [5], cone–rod dystrophy (CRD) [6], retinitis pigmentosa [7], generalized choriocapillaris dystrophy [8], and rapid onset chorioretinopathy [9].

The ABCA4 protein is a member of the superfamily of ATP-binding cassette (ABC) transporters and is predominantly localized to the rim region of rod and cone photoreceptor outer segment disk membranes [10]. It transports N-retinylidene-phosphatidylethanolamine (N-Ret-PE), a Schiff base adduct formed by retinal and phosphatidylethanolamine, from the lumen to the cytoplasmic leaflet of disk membranes. This process facilitates the efficient clearance of all-trans-retinal and excess 11-cis-retinal from photoreceptor cells, thereby preventing the buildup of toxic lipofuscin compounds within the RPE [11,12]. Cytotoxicity associated with A2E is thought to result in RPE dysfunction, ultimately causing progressive photoreceptor cell degeneration over time [13,14]. As a result, the accumulation of lipofuscin and bis-retinoid compounds is a hallmark feature of STGD1.

Understanding the phenotypic manifestations of STGD1 and identifying the causative genetic mutations are critical steps in unraveling its pathogenesis and can provide valuable insights for genetic counseling. The aim of this study was to delineate the comprehensive clinical and molecular genetic profiles of eighteen patients hailing from southeast China, all diagnosed with STGD1, as reported by a single center.

## 2. Results

### 2.1. Clinical Manifestations of the Patients

**Figure 1:**
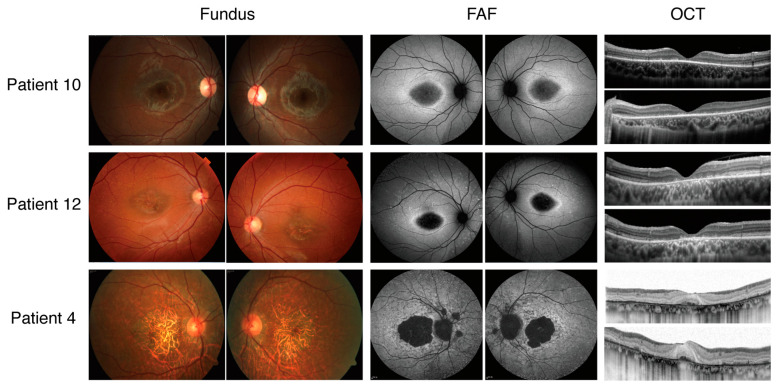
Color fundus appearances (**left**), fundus autofluorescence (FAF) images (**middle**), and optical coherence tomographic (OCT) images (**right**) from three representative cases of STGD1. Patient 10: The fundus image reveals central atrophy without flecks at the posterior; FAF imaging demonstrates a central area of reduced AF signals at the fovea with a homogenous background; OCT imaging shows the macular disruption of the outer retinal layers. Patient 12: The fundus image displays central atrophy surrounded by flecks localized to the posterior pole. FAF imaging identifies a central region of reduced AF signals at the macula, accompanied by numerous hyperautofluorescent foci at the posterior pole. OCT imaging reveals outer retinal disruption at the macula. Patient 4: The fundus image highlights extensive atrophic changes in the retinal pigment epithelium (RPE). FAF imaging reveals several areas of reduced AF signals at the posterior pole with a heterogeneous background. OCT images demonstrate widespread outer retinal disruption.

There were 18 patients with *ABCA4*-related Stargardt disease, among whom 6 exhibited adult onset (mean age of onset: 44.3; 3 females, 50%) and 12 exhibited onsets during childhood or adolescence (mean age of onset: 9.6; 6 females, 50%). Late-onset Stargardt disease represents a distinct subtype of Stargardt disease, characterized by its onset at the age of 45 years or later. In this study, four patients exhibited late-onset Stargardt disease. The age of examination ranged from 6 to 57 years and the duration of the disease ranged from 0 to a few decades. The patients all originated from the southeastern region of China. The mean best-corrected visual acuity (logMAR) was 0.96 for the right eye and 0.91 for the left eye, with a range from −0.10 to 1.98. Only one patient demonstrated a BCVA (logMAR) better than 0 in at least one eye. The baseline characteristics, clinical features, and mutation status of these 18 patients are summarized in Table 1. Autofluorescence imaging was available for 14 patients: 4 patients (28.6%) exhibited a type 1 AF pattern, 6 patients (42.8%) exhibited a type 2 AF pattern, and 4 patients (28.6%) exhibited a type 3 AF pattern. Representative fundus photographs, autofluorescence images, and OCT scans for three patients are presented in Figure 1.

**Figure 2:**
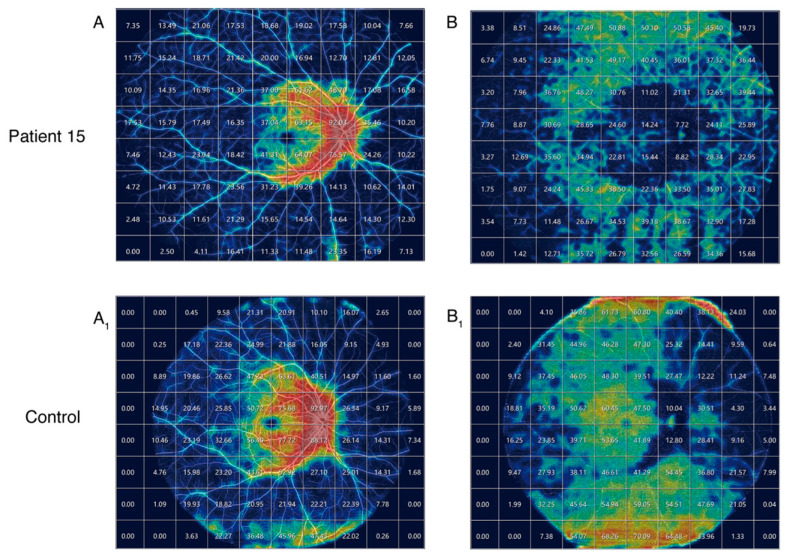
Quantification of superficial vascular complex (SVC) vessel density in Patient 15 compared to a normal control (**A**,**A_1_**) and the quantification of deep vascular complex (DVC) vessel density (**B**,**B_1_**). Red indicates high vascular density, while blue indicates low vascular density.

Patient 15 underwent ultra-widefield OCT angiography, with the OCTA images of 29 cm by 24 cm quantified using a grid size of 3 cm by 3 cm. Vessel density was represented by a heatmap (Figure 2). The superficial vascular complex (SVC) and the deep vascular complex (DVC) images were compared to those of a normal control subject (male aged 24). Compared to the control, no significant reductions in vessel density were observed in the superficial vascular complex. However, a marked decrease in vessel density was evident in the deep vascular complex surrounding the lesion.

### 2.2. Genetic Mutation Screening and Bioinformatic Analysis

We conducted the high-throughput sequencing of pathogenic genes associated with ocular genetic diseases in the peripheral blood DNA of all patients. Biallelic heterozygous ABCA4 variants were detected in 14 patients, while 3 patients (Patient 2, Patient 6, and Patient 15) had a homozygous variant and 1 (Patient 5) had three variants. In total, 31 ABCA4 variants were identified, including 11 missense, 6 nonsense, 4 splice-site alternations, 5 frameshifts, 4 intron variants, and 1 copy number variant. Moreover, eight distinct mutations from eight patients were novel mutations, including two nonsense variants (p.Val460Ter and p.Glu1058Ter), two frameshift deletions (p.Gln1852ArgfsTer3 and p.Leu2230ProfsTer17), one CNV (94480267-94482198del), one splice-site alternation (c.2919-1G>A), and two deep intron variants (c.1937+392G>A and c.2160+782T>C). No missense variants were located at the exon–intron boundaries and were predicted to cause splice-site alterations. The nonsense variants, frameshifts, and canonical ±1 or 2 splice-site variants were very strong pathogenic mutations. We employed the SpliceAI tool and the MaxEntScan online platform to predict the effects of four intronic variants that are not located at the canonical splicing sites (Table 2). SpliceAI predicted a donor loss with a high score (0.44) at position c.4253+5G>A and an acceptor loss at position c.6006-3C>A with a score of 0.42. The prediction suggested that these two mutations, c.1937+392G>A and c.2160+782T>C, do not affect splicing. The mutation c.1937+392G>A, identified in EnhancerDB, is situated within the enhancer vista2525 [chr1:94527681-94527931] and may potentially influence the enhancer’s regulation of ABCA4 gene expression.

### 2.3. Genotype–Phenotype Correlation

**Figure 3:**
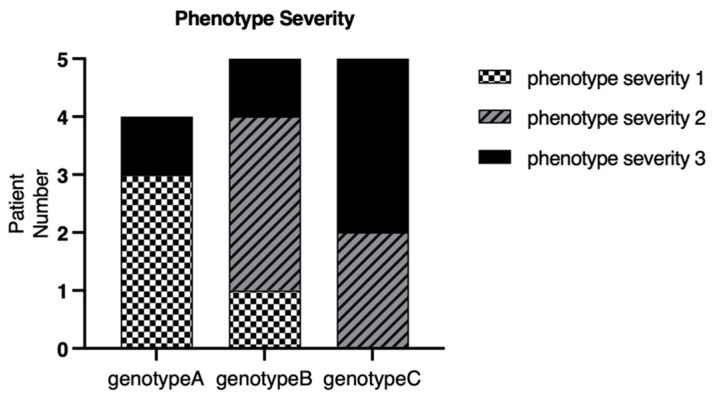
Phenotype severity classification for each genotype group.

After categorizing the patients with their mutation status, it was found that among the 18 patients, 5 were assigned to Group A (27.8%), 6 to Group B (33.3%), and 7 to Group C (38.9%). In the stratification by disease severity, six patients were categorized as severe (40.0%), five as moderate (33.3%), and four as mild (26.7%). The remaining three could not be classified due to incomplete clinical data. Patients with mutations categorized in Group C all exhibited childhood onset. For genotype A, the median age of onset was 39.4 years (range: 11–52), with a corresponding median BCVA (logMAR) of 0.748. In genotype B, the median age of onset was 18.5 years (range: 6–55), and the median BCVA (logMAR) was 0.878. For genotype C, the median age of onset was 10.4 years (range: 7–25), with a median BCVA (logMAR) of 1.113. The number of genotype A patients in the phenotype severity 1, 2, and 3 groups was three, zero, and one, respectively. The number of genotype B patients in the phenotype severity 1, 2, and 3 groups was one, three, and one, respectively. The number of genotype C patients in the phenotype severity 1, 2, and 3 groups was zero, two, and four, respectively (Figure 3).

**Figure 4:**
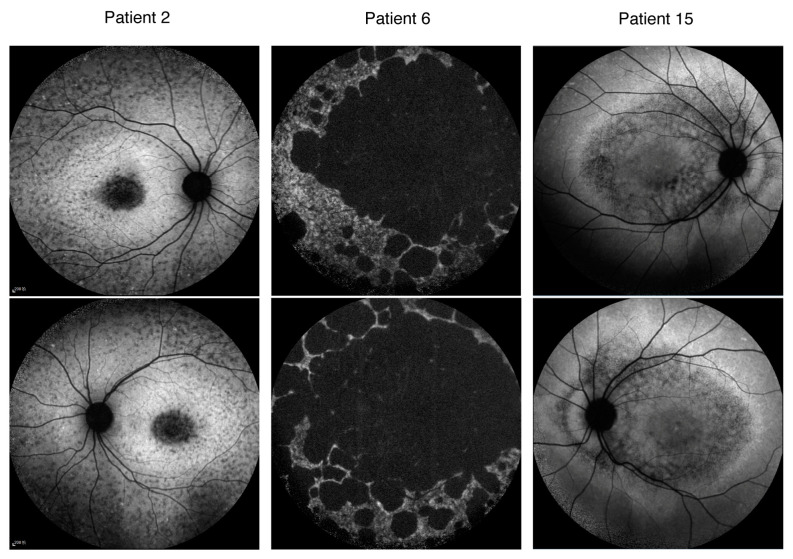
The fundus autofluorescence images from three patients with homozygous ABCA4 mutations: Patient 2 (c.6006-3C>A), Patient 6 (p.His1406Arg), and Patient 15 (c.2919-1G>A).

Three patients exhibited homozygous mutations in ABCA4, namely, Patient 2, Patient 6, and Patient 15. Patient 2 (p.6006-3C>A) began to experience unexplained vision loss at the age of 12, and by the age of 16, the BCVA (logMAR) in both eyes had dropped to only 1.30, with localized, low AF signals at the macula surrounded by a heterogeneous background in the AF image. Although Patient 6 (p.H1406R) presented with late onset in adulthood (at the age of 52), the disease progressed rapidly. The patient’s described medical history was only three months, yet the vision acuities had already deteriorated to FC/40cm (right eye) and FC/30cm (left eye). The patient exhibited multiple areas with reduced autofluorescence signals at the posterior pole of the fundus, with a heterogeneous background (type 3). Patient 15 harbored a mutation of c.2919-1G>A and experienced an onset of symptoms during childhood (<10 years). At the age of 13 years, the BCVA (logMAR) had reduced to 0.49 (right eye) and 0.60 (left eye). The fundus’s autofluorescence pattern was classified as type 3 (Figure 4).

## 3. Discussion

This study offers a comprehensive analysis of the clinical data and genetic characteristics of an STGD1 cohort consisting of 18 patients from southeast China. It also evaluates the genotype–phenotype correlations, identifying eight novel mutations in the ABCA4 gene.

Patients with late-onset Stargardt disease (onset after 45 years of age) typically exhibit a milder phenotype and slower disease progression [15,16]. In this study, four patients exhibited late-onset Stargardt disease. Excluding Patient 6, Patients 4, 16, and 17 all retained a BCVA value (logMAR) of less than 0.78.

The quantitative thermal maps of vascular density reveal a more pronounced reduction in Patient 15’s deep vascular complex (DVC) compared to the superficial vascular complex (SVC). In STGD1 patients, the primary atrophy of OCTA vasculature occurs in the choriocapillaris (CC), yet quantitative data on CC vascular density cannot be extracted from ultra-widefield OCTA.

Based on the genotype–phenotype correlation model by Cremers et al. in 1998 [17], in which the residual ABCA4 function inversely corresponds with disease severity, these patients with late-onset and mild symptoms typically had a genotype consisting of a combination of a mild and severe variant; no combinations of two severe variants were observed. It was noteworthy that Patients 16 and 17, who had exhibited late-onset symptoms, possessed a mutation pattern that included a missense mutation and a deep intronic mutation, and their vision was well preserved. This preservation of vision may have suggested that the deep intronic mutations they harbored had weaker pathogenicity or were nonpathogenic.

Consistent with a previous study [18], 31 ABCA4 variants identified in this study were distributed throughout the gene’s coding region. The most prevalent variant was c.2894A>G (p.Asn965Ser, 9.7%), differing from common variants (c.2424C>G, 4.7%, c.6563T>C, 3.7%, c.2894A>G, 3.1%, and c.101_106delCTTTAT, 3.1%) previously reported in Chinese Stargardt disease and cone–rod dystrophy cohorts [18]. The three most prevalent variants from the ProgStar study (c.5882G>A (p.Gly1961Glu); c.2588G>C (p.Gly863Ala); and c.5461-10T>C) were not observed here [19]. However, the p.Asn965Ser variant is the most frequent missense and founder-disease-associated ABCA4 variant in the Danish population [20] (16.2% of disease-associated alleles), and it is found sporadically in the American population. This fact indicated differences in genetic backgrounds between the Chinese and other populations.

Although over 4000 ABCA4 variants have been documented by Clinvar, recent research indicates that additional, previously unidentified disease-associated variants continue to be discovered in individuals with Stargardt disease (STGD1). In the present study, 31 variants were identified, with 8 of them being novel, including 2 nonsense variants (p.Val460Ter and p.Glu1058Ter), 2 frameshift deletions (p.Gln1852ArgfsTer3 and p.Leu2230ProfsTer17), 1 CNV(94480267-94482198del), 1 splice-site alternation (c.2919-1G>A), and 2 deep intron variants (c.1937+392G>A and c.2160+782T>C). The findings revealed that 31 variants were scattered across 21 exons and 8 introns of the ABCA4 gene, with 8 of these novel variants located in 5 exons and 3 introns. It is noteworthy that none of these eight novel mutations are missense mutations, which comprise the most common type of mutations in ABCA4, accounting for over 40% of all ABCA4 mutations. It is possible that advancements in genetic testing methods have enabled the detection of deep intronic mutations, which may also indicate that the genetic background of the population cohort in this study is different.

**Figure 5:**
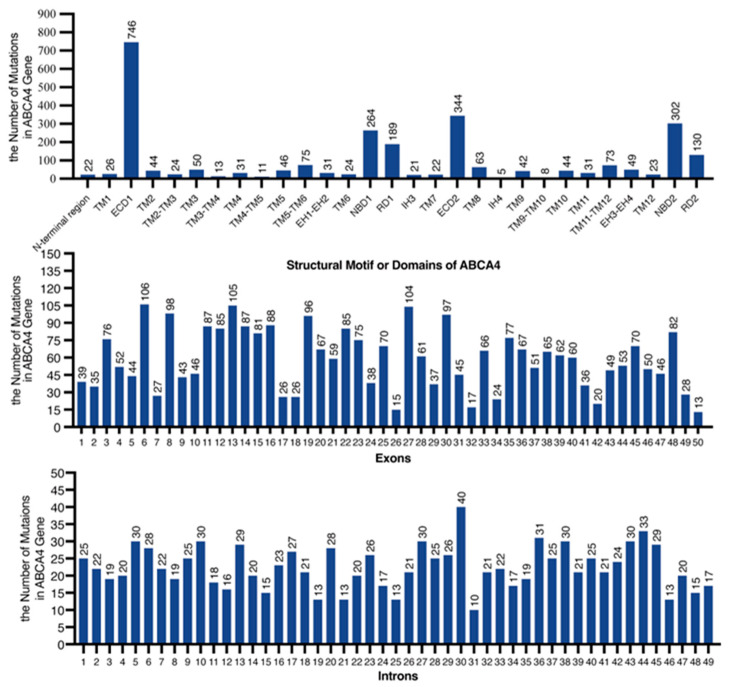
Mutation counts in ABCA4 protein domains, exons, and introns.

Additionally, we have conducted a statistical analysis based on the ClinVar database (https://www.ncbi.nlm.nih.gov/clinvar/, accessed on 19 November 2024) to determine the total number of reported mutations occurring in the exons of the ABCA4 gene that correspond to the protein domains of ABCA4. Mutations occurring in the ECD1 domain are the most numerous, followed by those in ECD2, NBD2, and NBD1, which is consistent with the molecular weight size of each domain. Additionally, we have also quantified the number of mutations occurring in the 50 exons and 49 introns of the ABCA4 gene (Figure 5). The top three are exons 6, 13, and 27; and introns 30, 44, and 36.

There are several limitations in this study. First, this observational cross-sectional retrospective case series study did not include longitudinal data. Additionally, the study was constrained by a small sample size, which precluded the execution of statistical analyses to ascertain the relationships between clinical parameters and specific genetic variants. Therefore, larger cohort studies are required for further detailed analyses of genotype–phenotype associations.

## 4. Materials and Methods

### 4.1. Participants and Clinical Investigations

The initial screening cohort comprised 512 patients derived from the 2017–2024 genetic screening database of the Zhongshan Ophthalmic Center for suspected inherited retinal diseases. Within this population, 352 cases were molecularly confirmed as retinal dystrophy (including 24 Stargardt disease diagnoses). The final analytical cohort consisted of 18 geographically restricted patients from southeast China, representing 3.52% of the confirmed retinal dystrophy cases in this institutional dataset. All 18 patients originated from southeast China. Clinical and molecular genetic diagnoses were confirmed by two senior doctors (YL and XL). Patients with additional ocular conditions, including choroidal neovascularization, glaucoma, and diabetic retinopathy, were excluded from the study. The patients underwent complete ophthalmic examinations, including best-corrected visual acuity (BCVA) examinations, dilated ophthalmoscopy, color fundus photography (Heidelberg Retina Angiograph, Heidelberg, Germany), fundus autofluorescence imaging (field of view: 55° × 55°; Spectralis, Heidelberg Engineering, Heidelberg, Germany), and flash ERG (Roland Consult, Brandenburg, Germany/LKC Technologies, Gaithersburg, MD, United States). Ultra-widefield OCT angiography was performed using SVision’s high-end SS-OCT product VG200D (Intalight, Luoyang, China), with an ultra-fast scanning speed of 200,000 A-scans per second, a wide field of view of 150 degrees, an imaging depth of 6.5 mm (in tissue), and a resolution of 1536 × 1280 pixels. Best-corrected visual acuity (BCVA) was converted into a logarithm of the minimum angle of resolution (LogMAR) units. Low vision categories, such as counting fingers (CFs) and hand motion (HM), were assigned values of 1.98 and 2.28, respectively [21]. Venous blood samples from the patients were collected.

### 4.2. Fundus Autofluorescence Type Classifications

The classification of fundus autofluorescence (AF) patterns was performed based on the criteria outlined by Fujinami K [2], dividing patients into three AF subtypes:

AF 1: Localized, low AF signals at the fovea surrounded by a homogeneous background, with/without perifoveal foci of high or low AF signals; AF 2: localized, low AF signals at the macula surrounded by a heterogeneous background and widespread foci of high or low AF signals extending anterior to the vascular arcades; AF 3: multiple areas of low AF signals at the posterior pole with a heterogeneous background, with/without foci of high or low AF signals.

### 4.3. Mutation Classifications

Previous studies categorized ABCA4 variants into three types: “mild”, “moderate”, and “severe”. The combinations of two allelic variants were shown to result in varying phenotypic outcomes [22,23]. According to Fujinami K, patients harboring ≥ 2 mutations were classified into three genotype groups based on the mutation type: Group A included individuals carrying two or more missense mutations or in-frame insertion/deletion variants; Group B encompassed patients with one deleterious mutation alongside one or more missense or in-frame insertion/deletion variants; Group C consisted of individuals harboring two or more definitely or likely deleterious (severe) mutations [24]. Null variants were those that would be expected to be very strong evidence of pathogenicity according to the standards and guidelines for the interpretation of sequence variants of ACMG, such as nonsense, frameshift, canonical ±1 or 2 splice sites, initiation codons, and single or multi-exonic deletions. Furthermore, one disease-associated intronic mutation with an unclear functional impact was classified as deleterious due to its association with a severe clinical phenotype reported in prior studies [25].

### 4.4. Classification of Severity for the Phenotype of Stargardt Disease

The classification of severity for the phenotype of Stargardt disease was performed based on clinical findings: age of onset, best-corrected visual acuity, fundus appearance, AF pattern, and electrophysiologic pattern (Table 3) [26].

### 4.5. Whole-Exome Sequencing and Sanger Sequencing of the ABCA4 Gene

A total volume of 15 mL of venous blood samples from the patients was collected. Whole-exome sequencing, variant verification, and bioinformatic analyses were performed following the protocol described in previous studies. Genomic DNA from peripheral blood leucocytes was extracted using the QIAamp DNABlood Midi kit (Cat. no. 51104, Qiagen GmbH, Hilden, Germany), following the manufacturer’s instructions. The genomic DNA was fragmented into approximately 200 base-pair (bp) fragments using Bioruptor Plus (Diagenode, Liege, Belgium). The generation of a paired-end library of whole exomes and library capture was completed using the xGen Exome Hyb Panel v1 kit (Integrated DNA Technologies, Inc., Shanghai, China). The library was sequenced with an MGISEQ-2000RS High-throughput Sequencing Set (PE150) on an MGISEQ-2000RS analyzer (MGI Tech Co., Ltd., Shenzhen, China). The average depth of the target regions was 100×, and the depth of over 95% of the target regions was more than 20×. A total of 42 probes targeting deep intron variants were added to our ES panel (NEXome Plus Panel version 1.0; Nanodigmobio, Guangzhou, China). Read alignment was performed using the human genome assembly hg19 and the Burrows–Wheler Aligner software (https://bio-bwa.sourceforge.net/, accessed on 5 October 2024). The detection of single-nucleotide polymorphisms (SNPs) and small insertions and deletions (indels) (<50 bp) was performed using GATK3.8 software. Sanger sequencing was applied to verify the suspected point mutations of the ABCA4 gene with standard procedures. Sequencing results were compared to the reference cDNA sequence of ABCA4 (GenBank NM_000350).

### 4.6. In Silico Molecular Genetic Analyses

All SNVs were annotated and filtered using databases, including the Human Genome Mutation Database (Professional Version, Qiagen, Germany), Leiden Open Variation Database (available at https://www.lovd.nl, accessed on 19 November 2024), and ClinVar (available at https://www.ncbi.nlm.nih.gov/clinvar, accessed on 19 November 2024). In silico tools were employed to predict the protein functional changes in missense mutations, including SIFT (available at http://sift.jcvi.org/, accessed on 19 November 2024), Polyphen-2 (available at http://genetics.bwh.harvard.edu/pph2/, accessed on 19 November 2024), PANTHER (available at https://www.pantherdb.org/, accessed on 19 November 2024), and SNP&GO (available at http://snps.biofold.org/snps-and-go/, accessed on 19 November 2024). The potential impact of intronic sequence variants on splice-site creation or disruption was evaluated using the SpliceAI tool [27] (available at https://spliceailookup.broadinstitute.org/, accessed on 19 November 2024) and MaxEntScan [28,29] (available at http://hollywood.mit.edu/burgelab/maxent/Xmaxentscan_scoreseq.html, accessed on 19 November 2024). Variants were filtered based on the SpliceAI delta score (range: 0–1), with a score of ≥0.2 indicating predicted pathogenicity. Additionally, for variants located within a functional donor or acceptor splice site, a ΔMaxEnt value (calculated as MaxEntvariant − MaxEntreference) of less than zero (ΔMaxEnt < 0) was considered indicative of a loss of the potential splice site. The classification of potentially pathogenic variants was performed in accordance with the guidelines set forth by the American College of Medical Genetics and Genomics and the Association for Molecular Pathology (ACMG) [30].

## 5. Conclusions

In conclusion, our study offers an in-depth analysis of the clinical profiles and genetic traits of a Chinese cohort comprising 18 patients from southeast China, uncovering eight novel ABCA4 gene mutations that were not previously documented. Furthermore, we have identified commonalities in the genotype–phenotype correlations. These insights contribute to a more nuanced understanding of the clinical and genetic landscape of Stargardt disease.

## Figures and Tables

**Table 1:** Summary of clinical features and genetic mutations of 18 patients with STGD1.

Pt. No	Sex	Age at Onset	Age	BCVA (logMAR) OD OS		Mutation Status	Mutation Group	Phenotype Severity	AF Pattern
1	F	7	8	1.40	1.30	c.5554del(p.Gln1852ArgfsTer3) *	C	3	1
						c.260dup(p.Gly88ArgfsTer11)			
2	F	12	16	1.30	1.30	c.6006-3C>A	C	2	2
3	F	7	8	1.12	1.12	c.4853G>A:(p.Trp1618Ter)	C	3	1
						chr1:94480267-94482198del *			
4	M	55	57	0.40	0.15	c.1378del(p.Val460Ter) *	B	1	3
						c.71G>A:(p.Arg24His)			
5	F	7	13	1	1	c.4253+5G>A	C	3	N/A
						c.3172G>T(p.Glu1058Ter) *			
						c.1531C>T(p.Arg511Cys)			
6	M	52	52	1.98	1.98	c.4217A>G(p.His1406Arg)	A	3	3
7	F	8	9	0.92	1	c.4217A>G(p.His1406Arg)	B	2	2
						c.1561del(p.Val521SerfsTer47)			
8	M	11	13	0.70	0.82	c.5761G>A(p.Val1921Met)	A	1	1
						c.1804C>T(p.Arg602Trp)			
9	M	11	31	1	1	c.2894A>G(p.Asn965Ser)	B	2	2
						c.2424C>G(p.Tyr808Ter)			
10	M	6	6	1.15	1	c.5646G>A(p.Met1882Ile)	B	3	1
						c.1761-2A>G			
11	M	25	25	1.10	1	c.1006del(p.Ser336ProfsTer38)	C	N/A	N/A
						c.303-2A>G			
12	M	17	17	1	0.92	c.2894A>G(p.Asn965Ser)	B	2	2
						c.1222C>T(p.Arg408Ter)			
13	F	14	19	1	1	c.3385C>T(p.Arg1129Cys)	B	N/A	N/A
						c.858+2T>A			
14	F	41	41	0	0.10	c.2894A>G(p.Asn965Ser)	A	1	N/A
						c.1531C>T(p.Arg511Cys)			
15	M	7	13	0.49	0.60	c.2919-1G>A *	C	2	2
16	F	46	47	0.40	0.60	c.42C>A(p.Asn14Lys)	A	1	2
						c.1937+392G>A *			
17	F	47	50	0.60	0.49	c.6119G>A(p.Arg1040Gln)	A	1	3
						c.2160+782T>C *			
18	M	8	15	1.70	1.15	c.6689del(p.Leu2230ProfsTer17) *	C	3	3
						c.2633C>A(p.Ser878Ter)			

The newly identified mutation is denoted by an asterisk (*).

**Table 2:** Predictions generated via SpliceAI and MaxEntScan.

Variant	Type	SpliceAI Delta Score	Position	ΔMaxEntScan
c.6006-3C>A ⇒1:94471141 G>T	Acceptor Loss	0.42 *	−3 bp	−3.702
c.4253+5G>A ⇒1:94496547 C>T	Donor Loss	0.44 *	+5 bp	−4.809
c.1937+392G>A ⇒1:94527741 C>T	N/A	0.01	−4 bp	N/A
c.2160+782T>C ⇒1:94525311 A>G	N/A	0.00	N/A	N/A

* The splice predicting tool SpliceAI delta score ≥ 0.02 (range: 0–1; predicted pathogenic ≥ 0.2), ΔMaxEntScan < 0 means the variants within a known functional donor or acceptor sequence of a splice site were defined as a loss of a potential donor or an acceptor splice site.

**Table 3:** Classification of severity for the phenotype of Stargardt disease.

	Age of Onset (yrs)	BCVA in the Better Eye (logMAR)	Fundus Appearance	AF Type	ERG Type
Mild (Group 1)	Later onset (>15)	<0.78	1	1	1
Moderate (Group 2)	Patients who did not meet at least 2 criteria of either mild or severe phenotype				
Severe (Group 3)	Early onset (<10)	>1.0	3	3	3

## Data Availability

The analyzed datasets generated during the study are available from the corresponding author upon reasonable request.

## References

[1] Michaelides M., Hunt D.M., Moore A.T. (2003). The genetics of inherited macular dystrophies. J. Med. Genet..

[2] Fujinami K., Lois N., Mukherjee R., McBain V.A., Tsunoda K., Tsubota K., Stone E.M., Fitzke F.W., Bunce C., Moore A.T. (2013). A longitudinal study of Stargardt disease: Quantitative assessment of fundus autofluorescence, progression, and genotype correlations. Investig. Ophthalmol. Vis. Sci..

[3] Gill J.S., Georgiou M., Kalitzeos A., Moore A.T., Michaelides M. (2019). Progressive cone and cone-rod dystrophies: Clinical features, molecular genetics and prospects for therapy. Br. J. Ophthalmol..

[4] Allikmets R., Singh N., Sun H., Shroyer N.F., Hutchinson A., Chidambaram A., Gerrard B., Baird L., Stauffer D., Peiffer A. (1997). A photoreceptor cell-specific ATP-binding transporter gene (ABCR) is mutated in recessive Stargardt macular dystrophy. Nat. Genet..

[5] Tanna P., Strauss R.W., Fujinami K., Michaelides M. (2017). Stargardt disease: Clinical features, molecular genetics, animal models and therapeutic options. Br. J. Ophthalmol..

[6] Maugeri A., Klevering B.J., Rohrschneider K., Blankenagel A., Brunner H.G., Deutman A.F., Hoyng C.B., Cremers F.P. (2000). Mutations in the ABCA4 (ABCR) gene are the major cause of autosomal recessive cone-rod dystrophy. Am. J. Hum. Genet..

[7] Martinez-Mir A., Paloma E., Allikmets R., Ayuso C., Del R.T., Dean M., Vilageliu L., Gonzalez-Duarte R., Balcells S. (1998). Retinitis pigmentosa caused by a homozygous mutation in the Stargardt disease gene ABCR. Nat. Genet..

[8] Bertelsen M., Zernant J., Larsen M., Duno M., Allikmets R., Rosenberg T. (2014). Generalized choriocapillaris dystrophy, a distinct phenotype in the spectrum of ABCA4-associated retinopathies. Investig. Ophthalmol. Vis. Sci..

[9] Tanaka K., Lee W., Zernant J., Schuerch K., Ciccone L., Tsang S.H., Sparrow J.R., Allikmets R. (2018). The Rapid-Onset Chorioretinopathy Phenotype of ABCA4 Disease. Ophthalmology.

[10] Molday R.S., Garces F.A., Scortecci J.F., Molday L.L. (2022). Structure and function of ABCA4 and its role in the visual cycle and Stargardt macular degeneration. Prog. Retin. Eye Res..

[11] Xu T., Molday L.L., Molday R.S. (2023). Retinal-phospholipid Schiff-base conjugates and their interaction with ABCA4, the ABC transporter associated with Stargardt disease. J. Biol. Chem..

[12] Al-Khuzaei S., Broadgate S., Foster C.R., Shah M., Yu J., Downes S.M., Halford S. (2021). An Overview of the Genetics of ABCA4 Retinopathies, an Evolving Story. Genes.

[13] Chen Y., Okano K., Maeda T., Chauhan V., Golczak M., Maeda A., Palczewski K. (2012). Mechanism of all-trans-retinal toxicity with implications for stargardt disease and age-related macular degeneration. J. Biol. Chem..

[14] Maeda A., Maeda T., Golczak M., Palczewski K. (2008). Retinopathy in mice induced by disrupted all-trans-retinal clearance. J. Biol. Chem..

[15] Westeneng-van H.S., Boon C.J., Cremers F.P., Hoefsloot L.H., den Hollander A.I., Hoyng C.B. (2012). Clinical and genetic characteristics of late-onset Stargardt’s disease. Ophthalmology.

[16] Fujinami K., Sergouniotis P.I., Davidson A.E., Wright G., Chana R.K., Tsunoda K., Tsubota K., Egan C.A., Robson A.G., Moore A.T. (2013). Clinical and molecular analysis of Stargardt disease with preserved foveal structure and function. Am. J. Ophthalmol..

[17] Cremers F.P., van de Pol D.J., van Driel M., den Hollander A.I., van Haren F.J., Knoers N.V., Tijmes N., Bergen A.A., Rohrschneider K., Blankenagel A. (1998). Autosomal recessive retinitis pigmentosa and cone-rod dystrophy caused by splice site mutations in the Stargardt’s disease gene ABCR. Hum. Mol. Genet..

[18] Jiang F., Pan Z., Xu K., Tian L., Xie Y., Zhang X., Chen J., Dong B., Li Y. (2016). Screening of ABCA4 Gene in a Chinese Cohort with Stargardt Disease or Cone-Rod Dystrophy With a Report on 85 Novel Mutations. Investig. Ophthalmol. Vis. Sci..

[19] Fujinami K., Strauss R.W., Chiang J.P., Audo I.S., Bernstein P.S., Birch D.G., Bomotti S.M., Cideciyan A.V., Ervin A.M., Marino M.J. (2019). Detailed genetic characteristics of an international large cohort of patients with Stargardt disease: ProgStar study report 8. Br. J. Ophthalmol..

[20] Rosenberg T., Klie F., Garred P., Schwartz M. (2007). N965S is a common ABCA4 variant in Stargardt-related retinopathies in the Danish population. Mol. Vis..

[21] Lange C., Feltgen N., Junker B., Schulze-Bonsel K., Bach M. (2009). Resolving the clinical acuity categories “hand motion” and “counting fingers” using the Freiburg Visual Acuity Test (FrACT). Graefe’s Arch. Clin. Exp. Ophthalmol..

[22] Cremers F., Lee W., Collin R., Allikmets R. (2020). Clinical spectrum, genetic complexity and therapeutic approaches for retinal disease caused by ABCA4 mutations. Prog. Retin. Eye Res..

[23] Tian L., Chen C., Song Y., Zhang X., Xu K., Xie Y., Jin Z.B., Li Y. (2022). Phenotype-Based Genetic Analysis Reveals Missing Heritability of ABCA4-Related Retinopathy: Deep Intronic Variants and Copy Number Variations. Investig. Ophthalmol. Vis. Sci..

[24] Fujinami K., Zernant J., Chana R.K., Wright G.A., Tsunoda K., Ozawa Y., Tsubota K., Robson A.G., Holder G.E., Allikmets R. (2015). Clinical and molecular characteristics of childhood-onset Stargardt disease. Ophthalmology.

[25] Zernant J., Schubert C., Im K.M., Burke T., Brown C.M., Fishman G.A., Tsang S.H., Gouras P., Dean M., Allikmets R. (2011). Analysis of the ABCA4 gene by next-generation sequencing. Investig. Ophthalmol. Vis. Sci..

[26] Fujinami K., Sergouniotis P.I., Davidson A.E., Mackay D.S., Tsunoda K., Tsubota K., Robson A.G., Holder G.E., Moore A.T., Michaelides M. (2013). The clinical effect of homozygous ABCA4 alleles in 18 patients. Ophthalmology.

[27] de Sainte A.J., Filser M., Isidor B., Besnard T., Gueguen P., Perrin A., Van Goethem C., Verebi C., Masingue M., Rendu J. (2023). SpliceAI-visual: A free online tool to improve SpliceAI splicing variant interpretation. Hum. Genom..

[28] Eng L., Coutinho G., Nahas S., Yeo G., Tanouye R., Babaei M., Dork T., Burge C., Gatti R.A. (2004). Nonclassical splicing mutations in the coding and noncoding regions of the ATM Gene: Maximum entropy estimates of splice junction strengths. Hum. Mutat..

[29] Shamsani J., Kazakoff S.H., Armean I.M., McLaren W., Parsons M.T., Thompson B.A., O’Mara T.A., Hunt S.E., Waddell N., Spurdle A.B. (2019). A plugin for the Ensembl Variant Effect Predictor that uses MaxEntScan to predict variant spliceogenicity. Bioinformatics.

[30] Richards S., Aziz N., Bale S., Bick D., Das S., Gastier-Foster J., Grody W.W., Hegde M., Lyon E., Spector E. (2015). Standards and guidelines for the interpretation of sequence variants: A joint consensus recommendation of the American College of Medical Genetics and Genomics and the Association for Molecular Pathology. Genet. Med..

